# CSBB: synthetic biology research at Newcastle University

**DOI:** 10.1042/BST20160437

**Published:** 2017-06-15

**Authors:** Angel Goñi-Moreno, Anil Wipat, Natalio Krasnogor

**Affiliations:** 1 Interdisciplinary Computing and Complex BioSystems (ICOS), School of Computing Science, Newcastle University, Newcastle upon Tyne NE1 7RU, U.K.; 2 Centre for Synthetic Biology and the Bioeconomy (CSBB), Newcastle University, Newcastle upon Tyne NE2 4AX, U.K.

**Keywords:** multidisciplinary, Newcastle, synthetic biology

## Abstract

The Centre for Synthetic Biology and the Bioeconomy (CSBB) brings together a far-reaching multidisciplinary community across all Newcastle University's faculties — Medical Sciences, Science, Agriculture and Engineering, and Humanities, Arts and Social Sciences. The CSBB focuses on many different areas of Synthetic Biology, including bioprocessing, computational design and *in vivo* computation, as well as improving understanding of basic molecular machinery. Such breadth is supported by major national and international research funding, a range of industrial partners in the North East of England and beyond, as well as a large number of doctoral and post-doctoral researchers. The CSBB trains the next generation of scientists through a 1-year MSc in Synthetic Biology.

## History of the centre

A fundamental component of the CSBB is the Interdisciplinary Computing and Complex Biosystems (ICOS) research group, which carries out groundbreaking research at the interface of computing science and complex biological systems. Jointly led by Natalio Krasnogor and Anil Wipat, the group has internationally recognised know-how in synthetic biology. Since the first participation in the iGEM competition (2008) to the organisation of events such as the latest IWBDA (International Workshop on Bio-Design Automation, 2016), members of the ICOS group are present in major activities within the field. As part of the community involvement, the group holds the chair of the Synthetic Biology Open Language (SBOL) — an open standard format for the exchange of designs of engineered biological systems. Additionally, ICOS leads the MSc in Synthetic Biology of the CSBB, which trains the new generation of synthetic biologists.

**Figure 1.**
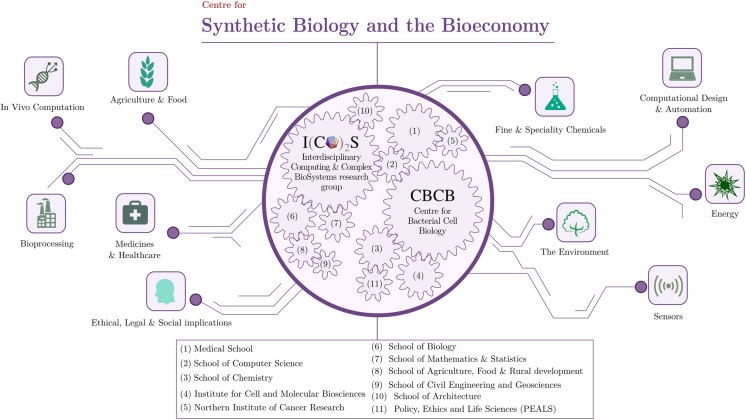
The Centre for Synthetic Biology and the Bioeconomy (CSBB) of Newcastle University. The CSBB brings together numerous academics across different faculties. The Interdisciplinary Computing and Complex Biosystems (ICOS) research group and the Centre for Bacterial Cell Biology (CBCB) play a relevant role in both governance and maintenance. The CSBB has established 10 research themes that cover all key areas in current synthetic biology. (Note: gears were located in no particular order).

ICOS activities are split between the School of Computing Science and the Centre for Bacterial Cell Biology (CBCB), a research centre of the Institute of Cell and Molecular Biosciences. The CBCB, directed by Jeff Errington, is the world's first major research centre with a focus on fundamental scientific questions about bacterial cells. In it, over 20 groups collectively address a wide range of bacterial-based problems from different angles, including synthetic biology approaches. In addition, there are other centres on campus, such as the Policy, Ethics and Life Sciences (PEALS) research centre, which are involved in different aspects synthetic biology through the efforts of many world-leading scientist. In such a scenario, the need to co-ordinate all efforts in synthetic biology emerged with clarity years ago. In 2012, Jeff Errington and Anil Wipat established the CSBB under the name Centre for Synthetic Biology and Bio-exploitation to bring together academics with expertise in synthetic biology that would traditionally be found in different faculties/schools. The CSBB was renamed Centre for Synthetic Biology and the Bioeconomy (http://www.ncl.ac.uk/csbb/) in 2015, upon the appointment of Natalio Krasnogor as its director (JE and AW co-direct the centre). Researchers of over 10 institutions across the University (Figure 1) constitute the core of what the CSBB is today. Interdisciplinary collaboration is encouraged through a regular series of talks by participants to highlight current research and potentiate the exchange of ideas. The recently launched Synthetic Portabolomics project (http://portabolomics.ico2s.org), a £7.5 million initiative jointly funded by the Engineering and Physical Sciences Research Council (EPSRC), Newcastle University and industry, is a good example of such collaboration within the CSBB. The Centre has been instrumental in securing Newcastle's inclusion in major synthetic biology initiatives across the U.K., such as the Flowers consortium and the SynbiCITE Innovation and Knowledge Centre. Synthetic biology research is rapidly growing at Newcastle as shown by the recent appointments of Tom Howard, Jon Marles-Wright and Dana Ofiteru in biochemistry and metabolic engineering, and Angel Goñi-Moreno in computational synthetic biology and mathematical modelling.

## Research themes

The CSBB is active in three broad categories within synthetic biology research: (i) new approaches, technologies and tools, (ii) applications and (iii) ethical, legal and social implications. The expertise available at the CSBB underpins the research carried out in Newcastle in many established themes (Figure 1).

- *Agriculture and Food*: Increasingly higher demands in food, water and energy are major challenges the world must face. These issues need to be addressed while at the same time combating poverty and preserving biodiversity and the environment. Synthetic biology offers a new framework in which to tackle these problems via crop engineering.
- *Bioprocessing*: While bioprocessing has been a successful mainstay of biotechnology for many years, major challenges still remain. One of them is the scaling up of lab-based systems to industrial production. The rational design of systems empowers synthetic biology with the capabilities to enhance the efficiency of such processes.
- *Computational design and automation*: An important objective of the CSBB is to aid researchers to transit from manual design to computer-aided design processes. Synthetic biologists can no longer rely on error-prone and time-consuming manual efforts. Software tools and data standards aim at turning design automated and reliable.
- *Energy*: Pollutant accumulation from the use of fossil fuels, such as carbon dioxide, will inevitably lead to global catastrophic environmental impact. The potential to engineer organisms to act as sustainable energy sources is one of the most attractive topics within synthetic biology. Researchers in this theme at the CSBB focus on various lines ranging from biofuels to the production of electricity by sunlight-sensitive engineered cells.
- *The environment*: A new wave of bioremediation strategies are strengthened owing to synthetic biology approaches. Nevertheless, the release of complex engineered organisms into the environment is still a technical challenge.
- *Ethical, legal and social implications of synthetic biology*: The internationally recognised PEALS Research Centre has collaborated for over 15 years with scientists, clinicians, policymakers and civil society groups. At the CSBB, it considers the benefits and potential risks of synthetic biology.
- *Fine and speciality chemicals*: Researchers in this area engineer model organisms to produce a variety of chemicals and chemical intermediates. The synthetic biology approach comes when a wide range of modelling and design techniques are combined with genomic-scale modifications to pursue predefined requirements.
- *In vivo computation*: Cells, as bacteria, can be conceptualised as machines that process information according to internal circuits to turn inputs into outputs. That internal circuitry can be built, from DNA ‘parts’, to mimic electronic devices. This research aims at programming bacteria to fulfil predefined computing requirements.
- *Medicines and healthcare*: Applications in this area include the development of new therapeutics and overcoming antibiotic resistance*.* Integration with other research themes will, in the long term, enable the design of bacteria programmed to target and intervene in cancerous cells.
- *Sensors*: Synthetic biology fostered the engineering of novel biosensors. Our ability to fine-tune bacterial sensing systems and use them alongside micro-device technologies allows for quantifiable outputs in real time. A wide range of applications are researched, depending on the nature of the signal being monitored e.g. medical diagnostics or environmental sensing.

## Resources and facilities

The main facilities are located at the Centre for Bacterial Cell Biology (CBCB). It has world-class installations for all synthetic biology areas, including state-of-the-art laboratories. Microscopy facilities are specialised in fluorescence imaging allowing a wide range of measurements, such as 3D imaging, 4D multi-position, FRAP analysis or flow cell imaging. As a large contributor to the CSBB, the School of Computing Science provides cluster availability for computer-intensive analysis of large data sets. The Centre also integrates robotic facilities, since modern high-throughput biology requires a high degree of automation. The CSBB continually strives to provide top-of-the-range facilities, which currently include an Ion Torrent PGM genome sequencer and robots for the large-scale analysis of variations in yeast colonies.

Microfluidics and lab-on-a-chip technologies are becoming a powerful tool for synthetic biology since they permit single-cell manipulation to an unprecedented extent. We built our microfluidics foundry with flexibility and scalability in mind, to ensure we can keep up with this rapidly evolving field.

The members of the Centre have produced a large number (over 20) of Software tools and resources for Synthetic Biology that have been released to the community. Details are available at http://www.ncl.ac.uk/csbb/research/software-data-repository-resources/ and http://ico2s.org/resources.html.

Over the last 5-year period, interdisciplinary funding secured by members of the CSBB amount to over £18 million. Key funding bodies contributing to such a figure include the Engineering and Physical Sciences Research Council (EPSRC), the Biotechnology and Biological Sciences Research Council (BBSRC), the Natural Environment Research Council (NERC), the European Union and industry collaborations among others.

## Competing Interests

The Authors declare that there are no competing interests associated with the manuscript.

